# Oxygen production as an electron overflow pathway in ammonia-oxidizing archaea

**DOI:** 10.1126/sciadv.aee5234

**Published:** 2026-08-14

**Authors:** Thomas Pribasnig, Christa Schleper, Logan H. Hodgskiss

**Affiliations:** Department of Functional and Evolutionary Ecology, Archaea Biology and Ecogenomics Unit, University of Vienna, Djerassiplatz 1, 1030 Vienna, Austria.

## Abstract

Ammonia-oxidizing archaea (AOA) are widespread in nature and contribute to global carbon and nitrogen cycling. Although their energy production is an aerobic process, AOA occur in environments of low oxygen and in fully anoxic zones. Recently, oxygen production under anoxic conditions was demonstrated, raising the hypothesis that this activity can sustain oxygen dependent ammonia oxidation. In the work presented here, the production of high amounts of oxygen by *Nitrososphaera viennensis* under anoxia is dependent on nitrite, but not ammonia. Rather than conferring a physiological benefit, oxygen production impaired recovery from anoxia. Produced oxygen is mostly not consumed and, therefore, cannot sustain ammonia oxidation in the absence of external oxygen. Instead, it is tightly linked to recent energetic activity. These observations indicate that oxygen production represents a general physiological strategy for alleviating the potential accumulation of reactive intermediates in the ammonia oxidation pathway, analogous to nitrifier denitrification in ammonia-oxidizing bacteria.

## INTRODUCTION

Ammonia-oxidizing archaea (AOA) gain energy by oxidizing ammonia to nitrite (*1*, *2*), through which they contribute to the global nitrogen cycle (*3*, *4*). AOA are efficient in their central metabolism. They use the most energy efficient aerobic carbon fixation cycle (*5*), and a type aa_3_ terminal oxidase as complex IV (*6*), the most efficient terminal oxidase in terms of protons pumped per electron (*7*, *8*). The ammonia monooxygenase (AMO) requires the input of two electrons from menaquinone to carry out the oxidation of NH_3_ to NH_2_OH (*9*–*11*), which is further oxidized to NO_2_^−^ by one or more unknown enzymes for energy conservation (*12*–*15*). AOA do not only have a high affinity for NH_3_ (*16*–*19*) but have also been shown to have a high affinity for oxygen, allowing activity under low oxygen concentrations (*20*). It may be their extremely efficient metabolism that enables their ubiquitous distribution in almost every aerobic environment (*21*–*23*). Unexpectedly, they have also been found to be highly abundant in environments with low or no oxygen (*24*–*28*); however, no measurable ammonia oxidation activity explicitly by AOA has been reported in the complete absence of oxygen. Their occurrence in those environments is unexpected, because AMO and the terminal oxidase both rely on oxygen for activity, making them dependent on oxygen for their metabolism.

The capability of *Nitrosopumilus maritimus*, and other AOA including the soil strain *Nitrososphaera viennensis*, to produce oxygen under anoxic conditions markedly changed that view (*29*, *30*). In this proposed pathway, NO_2_^−^ is first reduced to NO, presumably by the nitrite reductase (NirK), and subsequently dismutated to N_2_O and O_2_ in all evaluated strains (*30*). Independent from this pathway, N_2_O production by AOA has been demonstrated in several studies, either as a by-product of hydroxylamine oxidation or as a result of NO_2_^−^ reduction (*20*, *31*–*34*). Under anoxic conditions, a further reduction of N_2_O to N_2_ was found to be species specific (*30*, *35*). Whereas some strains, such as *N. viennensis*, stop at N_2_O, others, like *N. maritimus*, further reduce it to N_2_. Even the reduction of exogenous N_2_O has been described, making specific strains of AOA potential sinks for N_2_O under anoxic conditions (*35*). Oxygen production could expand the range of AOA activity into anoxic environments. However, oxygen production requires more electrons than can be obtained from ammonia oxidation alone (*29*). So far, all isolated AOA that have been shown to produce oxygen are strict autotrophs and have not been shown to gain electrons from any other source than ammonia oxidation (*1*, *32*, *36*, *37*). The further reduction of N_2_O to N_2_ is not compatible with the current interpretation of oxygen production as a method to sustain activity under anoxic conditions. This is in stark contrast to the otherwise extremely efficient metabolism used by AOA and the need to streamline expenses even further if activity should be sustained under anoxic conditions.

In this study, a range of physiological experiments was used to investigate oxygen production in the soil ammonia oxidizer, *N. viennensis*. Oxygen production is proposed as a NirK dependent electron overflow pathway rather than an adaptation to anoxic conditions. It is shown that produced oxygen cannot sustain ammonia oxidation in the absence of external oxygen and is instead tightly linked to recent energetic activity. Together, these findings reshape the view of oxygen production in AOA, positioning it as a general physiological strategy for addressing the potential buildup of reactive intermediates in the ammonia oxidation pathway rather than sustaining aerobic ammonia oxidation under anoxic conditions.

## RESULTS

### Oxygen production is dependent on NO_2_^−^ and independent from NH_4_^+^

**Fig. 1.**
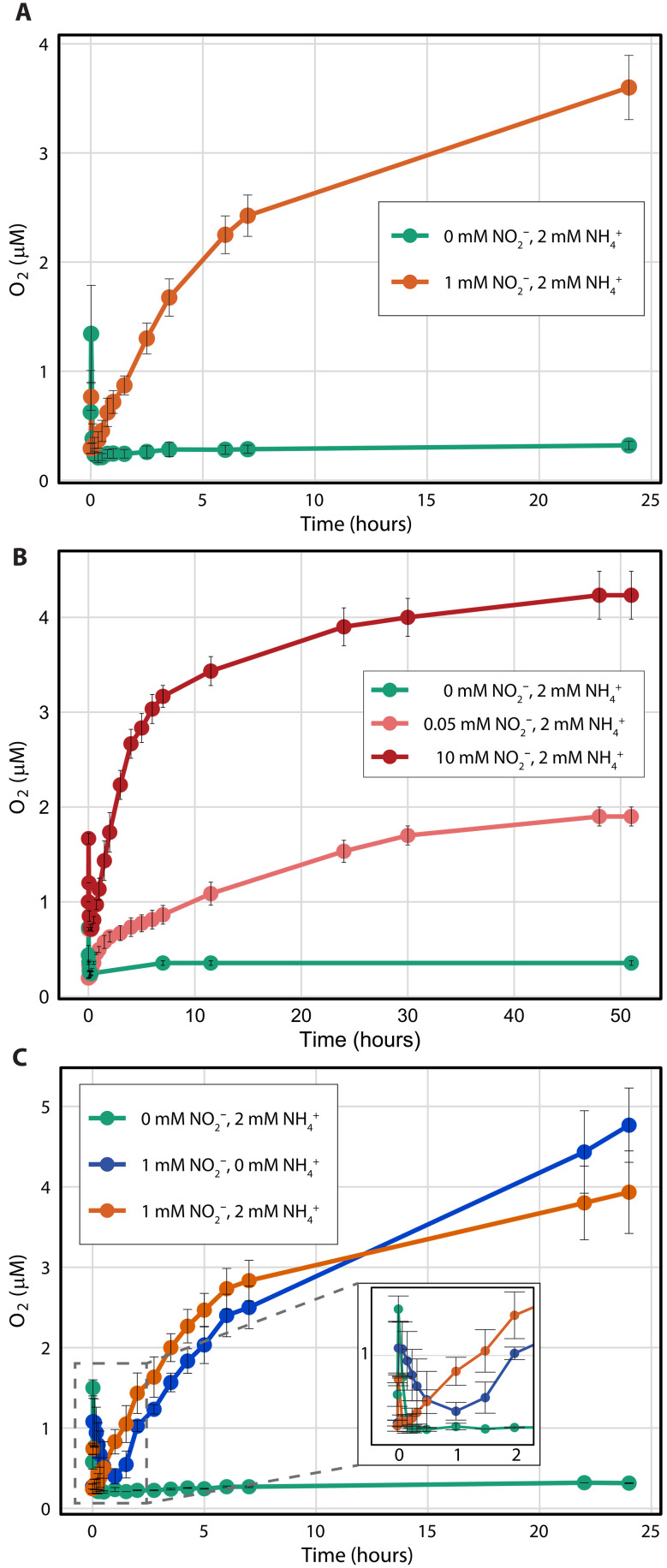
Oxygen production is dependent on NO_2_^−^ and independent from NH_4_^+^. Oxygen production [in micromolar (μM)] of *N. viennensis* under anoxic conditions over time (in hours). Conditions contain 2 mM NH_4_^+^ and 1 mM NO_2_^−^, unless otherwise specified. (**A**) Oxygen production with or without nitrite (*n* = 4 biological replicates). (**B**) Oxygen production with varying concentrations of nitrite (0.05 mM NO_2_^−^ and 10 mM NO_2_^−^, *n* = 3 biological replicates; 0 mM NO_2_^−^, *n* = 2 biological replicates). (**C**) Oxygen production with or without ammonium (with NO_2_^−^, *n* = 3 biological replicates; without NO_2_^−^, *n* = 2 biological replicates). Error bars represent SD for *n* ≥ 3 and max and min values when *n* = 2. Exact cell numbers and raw data can be found in dataset S1.

*N. viennensis* cells were washed to remove residual NO_2_^−^ and NH_4_^+^ and inoculated into a near-anoxic medium with or without NO_2_^−^ (Fig. 1A) or NH_4_^+^ (Fig. 1C) to initial concentrations of 6.12 × 10^7^ cells/ml. In both conditions, the introduced oxygen was rapidly consumed to ∼0.2 to 0.3 μM. In the presence of NO_2_^−^, oxygen concentrations increased immediately after reaching the minimum and continued increasing for 24 hours, with the highest production rates in the first hours and a gradual slowdown over time. The final oxygen concentration measured (3.5 μM) was an order of magnitude higher than reported in previous studies (*29*), due to the use of concentrated cells (∼5× the amount of cells/ml as previous studies (*29*), exact numbers for current study supplied in dataset S1; Fig. 1A). In the absence of NO_2_^−^, no increase in oxygen levels was observed after the initial depletion and oxygen levels stayed constant at ∼0.3 μM over 24 hours (Fig. 1A). Experiments with higher NO_2_^−^ resulted in larger concentrations of oxygen, reinforcing the dependence on NO_2_^−^ (Fig. 1B).

To test the dependence of oxygen production on supplied NH_4_^+^, cells were inoculated into a near-anoxic medium with 1 mM NO_2_^−^ containing either 2 or 0 mM NH_4_^+^, alongside a control with 0 mM NO_2_^−^ (Fig. 1C). In the presence of NH_4_^+^, oxygen was rapidly consumed to ∼0.3 μM. When NO_2_^−^ was present in the medium, oxygen concentrations increased again, as observed previously. Without NH_4_^+^, oxygen consumption was slower, reaching ∼0.3 μM after 1 hour. Based on the oxygen introduced with the inoculum (∼1 μM), only a limited amount of NH_3_ oxidation could be supported [corresponding to a maximum of ∼1 μM NH_3_ if all O_2_ is used by the AMO, or ∼0.7 μM when accounting for activity of the terminal oxidase, based on thermodynamic modeling; (*38*)]. While intracellular NH_4_^+^ storage in that magnitude would be possible and cannot be entirely excluded, the distinct consumption dynamics indicate that externally supplied NH_4_^+^ strongly influences the initial metabolic response, and any internal pools are likely minimal and/or rapidly depleted.

Regardless, with and without NH_4_^+^, oxygen concentrations increased immediately after a minimum was reached in the presence of NO_2_^−^, with similar production rates in both conditions. After 24 hours, the measured oxygen concentration in the presence of NH_4_^+^ and NO_2_^−^ was slightly higher than in conditions with only NO_2_^−^ (4.0 μM with NH_4_^+^ and 4.7 μM without NH_4_^+^), while the variation between replicates was of similar magnitude (Fig. 1C).

### Oxygen production does not convey a competitive advantage under fluctuating oxygen conditions

**Fig. 2.**
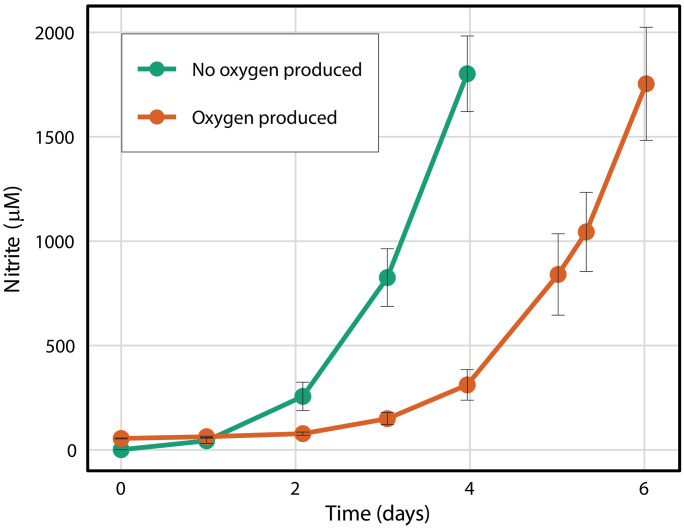
Aerobic recovery after 24 hours in anoxic conditions. Growth curves of *N. viennensis* in optimal oxic growth medium after either producing or not producing oxygen in anoxia (*n* = 12 biological replicates). Recovery was monitored by nitrite production [in micromolar (μM)] over time (in days). Error bars represent SD. Exact cell numbers and raw data can be found in dataset S1.

Recovery after 24 hours in anoxic conditions was tested to determine whether oxygen production would lead to a competitive advantage after anoxic conditions. The same cultures shown in Fig. 1A, which, after 24 hours of anoxic incubation, had either produced oxygen (incubated with 1 mM NO_2_^−^ and 2 mM NH_4_^+^) or not (incubated with 0 mM NO_2_^−^ and 2 mM NH_4_^+^), were returned to optimal oxic growth conditions. Recovery was substantially better for cells that had not produced oxygen than for those that had. Cultures that had produced oxygen showed an extended lag phase of 3 days, after which growth started at slightly lower rates than in the cultures that had not produced oxygen (Fig. 2). An effect of NO_2_^−^ toxicity can be excluded in all experiments, as *N. viennensis* has been shown to be insensitive to NO_2_^−^ concentrations of this magnitude (*39*, *40*).

### Oxygen production is dependent on recent energetic activity

**Fig. 3.**
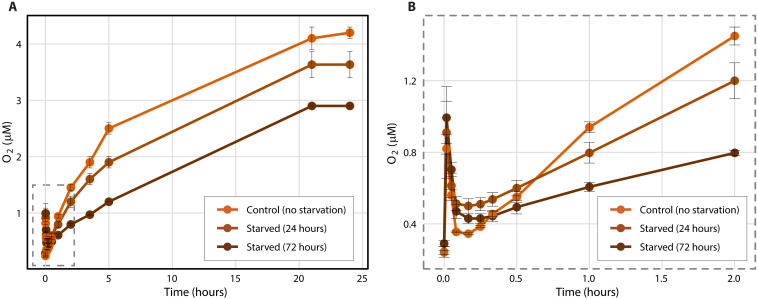
Oxygen production after starvation. Oxygen production [in micromolar (μM)] of *N. viennensis* under anoxic conditions over time (in hours). All cultures were supplied with 2 mM NH_4_Cl and 1 mM NO_2_^−^ after starvation. (**A**) Oxygen production after ammonium starvation in oxic conditions (starved cultures, *n* = 3 biological replicates; control, *n* = 2 biological replicates). (**B**) A zoomed-in view of the dashed boxed in (A) showing the first 2 hours to display different consumption dynamics due to starvation effects. Error bars represent SD for *n* ≥ 3 and max and min values when *n* = 2. Exact cell numbers and raw data can be found in dataset S1.

A dependence of oxygen production on NO_2_^−^ would necessitate a supply of electrons from reduced electron carriers (i.e., blue copper proteins) to reduce NO_2_^−^ to NO, which is subsequently dismutated to O_2_ and N_2_O. To test whether the energetic state of cells influences the capacity and amount of oxygen production, cells were starved in normal oxic conditions before inoculation into-near anoxic medium containing 1 mM NO_2_^−^ and 2 mM NH_4_^+^. Direct inoculation (no starvation) resulted in the same oxygen consumption and production patterns observed previously. Starvation for 1 or 3 days led to reduced initial oxygen consumption, with a minimum of ∼0.5 μM compared to ∼0.3 μM without starvation (Fig. 3). In all cases, oxygen concentrations increased immediately after reaching the respective minimum, but initial rates (in the first 5 hours) were slower in starved cells, i.e., ammonia starvation reduced the capacity for oxygen production. Overall, starvation reduced both the initial production rates and the total amount of oxygen produced. After 24 hours, cells without prior starvation produced oxygen to a concentration of 4.3 μM, those starved for 1 day produced 3.6 μM, and cells starved for 3 days produced 2.9 μM (Fig. 3). These results show that the capacity for oxygen production depends on recent energetic activity of the cell and is likely linked to the availability of electrons from reducing equivalents (i.e., reduced blue copper proteins).

**Fig. 4.**
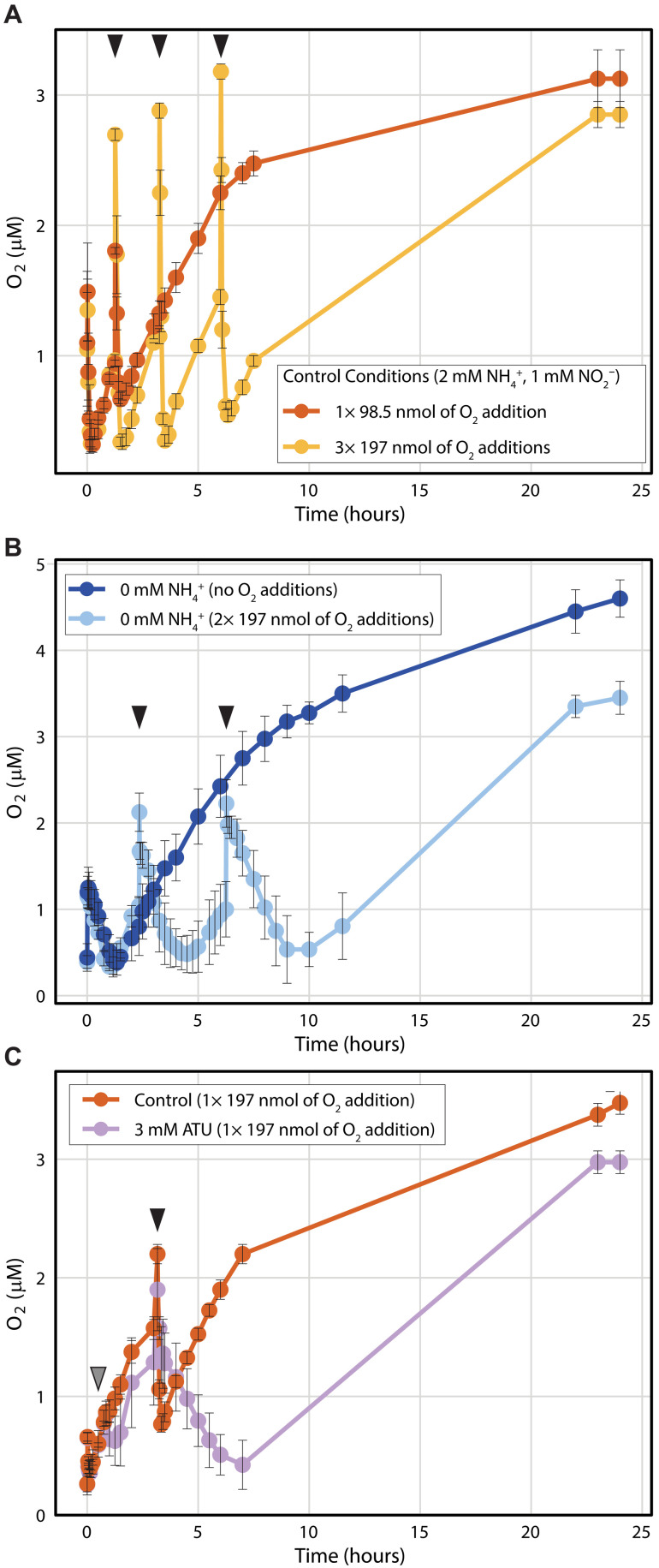
Oxygen production with additions of oxygen. Oxygen production [in micromolar (μM)] under anoxic condition over time (in hours). All cultures were supplied with 2 mM NH_4_Cl and 1 mM NO_2_^−^, unless otherwise specified. Black arrows indicate time of oxygen addition. (**A**) Production and consumption of oxygen by cultures of *N. viennensis* with the addition of external oxygen (*n* = 4 biological replicates). (**B**) Production and consumption of oxygen with 0 mM NH_4_^+^ by cultures of *N. viennensis* with the addition of external oxygen (*n* = 4 biological replicates). (**C**) Production and consumption of oxygen in the presence of ATU, a nitrification inhibitor, by cultures of *N. viennensis* with the addition of external oxygen (*n* = 4 biological replicates). Error bars represent SD. Gray arrow indicates the addition of ATU in anoxic medium or anoxic medium alone. Exact cell numbers and raw data can be found in dataset S1.

**Fig. 5.**
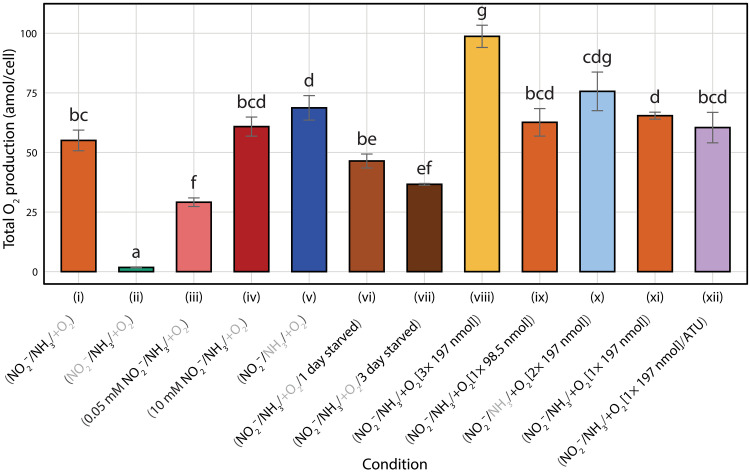
Oxygen accumulation over all experiments. Oxygen accumulation (in attomoles per cell) in 24 hours under different conditions. Figure labels indicate the presence of 2 mM NH_4_Cl, NO_2_^−^ (1 mM, unless otherwise stated), starvation, ATU, or oxygen additions in bold, or the absence in gray. Brackets after oxygen additions indicate the number of additions made and the amount of oxygen introduced by each addition in nanomole. Statistical significance between conditions is indicated by letters and determined by a Games-Howell test and an adjusted *P* using the Benjamini-Hochberg method. Error bars represent SD. Colors of bars correspond to colors in Figs. 1 to 4.

To further test the dependency of oxygen production on the availability of reduced blue copper proteins, the effect of adding external oxygen during oxygen production in nonstarved cultures was tested. An influx of oxygen would allow for the replenishment of reducing equivalents in the pseudo-periplasm (i.e., blue copper proteins). This would be achieved by activating the AMO complex for the transfer of electrons from electron carriers in the membrane (i.e., menaquinones) to produce hydroxylamine from ammonia and oxygen. This hydroxylamine could then be oxidized and electrons transferred to blue copper proteins. Cells were inoculated into medium containing 1 mM NO_2_^−^ and 2 mM NH_4_^+^ and divided into two groups. One group received a single oxygen addition of 98.5 nmol (increasing the concentration by 0.82 μM), whereas the other received three larger additions of 197 nmol each (increasing the concentration by 1.64 μM each time). Additions were made once cultures had produced ∼1 μM oxygen. In both cases, external oxygen introductions lead to oxygen consumption (Fig. 4A). The consumption of added oxygen was observed even in the case where added oxygen resulted in total oxygen levels below the oxygen production limit of the culture (Fig. 4A). The first two larger additions (increasing concentrations by 1.64 μM) were fully consumed, whereas the smaller addition (increasing concentrations by 0.82 μM) was only partially consumed, reaching a minimum concentration at ∼0.6 μM. When the last 1.64 μM addition was made at 1.5 μM oxygen, consumption again was only partial, reaching a minimum of ∼0.4 μM. Despite these differences in consumption patterns, the final oxygen concentration after 24 hours was the same between treatments (Fig. 4). However, total oxygen amounts, calculated from the sum of produced oxygen, were substantially higher in the group with multiple oxygen additions (Fig. 5).

The experiment was repeated without NH_4_^+^ in the medium (Fig. 4B). In the absence of ammonia, externally added oxygen should not replenish reducing equivalents due to AMO inactivity. As observed previously (Fig. 1B), initial oxygen consumption was slower, and concentrations increased after the minimum concentration of ∼0.3 μM was reached. One group was left undisturbed, whereas the other received two oxygen additions of 197 nmol (increasing the concentrations by 1.64 μM). Added oxygen was consumed slowly to ∼0.4 μM before production resumed. After 24 hours, undisturbed cultures reached an oxygen concentration of 4.5 μM, whereas cultures with additions reached only 3.5 μM (Fig. 4B) However, the total oxygen production, calculated from the sum of produced oxygen, was similar (Fig. 5). The experiment was repeated in the presence of NH_4_^+^ but with allylthiourea (ATU), a specific inhibitor of the AMO complex in AOA (*10*, *41*), added to inhibit the AMO complex in the presence of NH_4_^+^ (Fig. 4C). The presence of ATU resulted in slower oxygen consumption and production patterns identical to those observed in cultures without NH_4_^+^, indicating that the observed patterns were due to inactivity of AMO rather than solely an absence of NH_4_^+^.

Figure 5 summarizes the oxygen accumulation normalized to cell number observed across all experiments. Because exact production is unknown, oxygen accumulation is described. Over all experiments, standard oxygen production conditions (1 mM NO_2_^−^ and 2 mM NH_4_^+^) yielded 55 ± 4.1 amol cell^−1^ (*n* = 9) [Fig. 5 (i)]. Incubations without NO_2_^−^ did not accumulate significant amounts of oxygen (*n* = 8) [Fig. 5 (ii)]. Incubations with 0.05 mM NO_2_^−^ yielded a decreased amount of oxygen 29 ± 1.5 amol cell^−1^ (*n* = 3) [Fig. 5 (iii)], whereas incubations with 10 mM NO_2_^−^ yielded with 61 ± 3.3 amol cell^−1^ (*n* = 3) [Fig. 5 (iv)]. Incubations with NO_2_^−^ but without NH_4_^+^ led to a higher accumulation of 68.7 ± 4.7 amol cell^−1^ (*n* = 7) [Fig. 5 (v)]. When cells were starved, before incubations, accumulation was decreased to 46.4 ± 2.4 amol cell^−1^ (*n* = 3) after 1 day of starvation and to 36.6 ± 0.3 amol cell^−1^ (*n* = 3) after 3 days of starvation [Fig. 5 (vi and vii)]. In the presence of NH_4_^+^, additions totaling 591 nmol of oxygen (three additions of 197 nmol each, increasing the concentration by 1.64 μM each) led to an increased net accumulation of 98.7 ± 4 amol cell^−1^ (*n* = 4) [Fig. 5 (viii)], whereas a small addition of 98.5 nmol (increasing the concentration by 0.82 μM) led to a net accumulation of 62.6 ± 5 amol cell^−1^ (*n* = 4) [Fig. 5 (ix)]. In the absence of NH_4_^+^, additions of 394 nmol (two additions of 197 nmol each, increasing the concentration by 1.64 μM each) of oxygen [Fig. 5 (x)] led to an accumulation of 75.6 ± 7 amol cell^−1^ (*n* = 4). ATU treatment [Fig. 5 (xii)] led to similar patterns as conditions in the absence of NH_4_^+^ (x) and was similar to the control without ATU (xi) of 60.4 ± 5.5 amol cell^−1^ (*n* = 4) [Fig. 5 (xi and xii)].

### Transcriptomic changes under anoxic conditions

To analyze the change in transcriptomic landscape, transcriptomes were produced after 2 and 24 hours under anoxic conditions in two separate experiments in which cells had produced oxygen to a concentration of 1.2 or 3.4 μM, respectively. In each experiment, five replicates were analyzed for controls and anoxic cultures. Control samples were harvested directly from the same cultures under oxic conditions before transfer into near-anoxic medium and processed in parallel with the experimental samples. After 24 hours, the extracted RNA and resulting transcriptome showed clear signs of degradation (figs. S1 to S4). This resulted in a transcript-per-million (TPM) value of >10 for only the 250 most abundant transcripts (compared to 2886 for control samples). Additionally, the RNA pool after ribosomal RNA (rRNA) depletion was dominated by two noncoding RNA (ncRNA) genes. Ninety-five percent of all RNA corresponded to *NVIE_RNA1086407D* and *NVIE_RNA0139656D*, both of which are components of RNA-protein complexes (signal recognition particle and a ribonucleaseP, respectively).

This is substantially higher than for the controls for which these transcripts made up 10% of sequenced RNA. The mRNA corresponding to the respective proteins of these complexes was not highly expressed (TPM < 100), and, although mRNA and protein levels are not always positively correlated, this led to the assumption that the highly abundant ncRNA genes were not up-regulated but were rather protected from degradation and represented the only RNA left in an otherwise degraded transcriptome.

In a follow-up experiment, the same trends held true after 2 hours, but to a lesser degree, where 65% of all RNA corresponded to one of the two complexed ncRNAs, whereas none of the mRNAs corresponding to the associated proteins showed high abundance.

Across all replicates, consistent degradation was observed and found to be more extreme in prolonged anoxic conditions, indicating that this was not an isolated technical artifact but a reproducible outcome under anoxic conditions. Control samples processed in parallel did not show comparable degradation (figs. S1 to S4), despite identical RNA extraction (and comparable levels of RNA yields, dataset S1), library preparation, and sequencing procedures. Signs of degradation could also be viewed in quality control data of extracted RNA (figs. S1 to S4). Due to the global shifts in transcriptomes based on degradation, a differential expression analysis was not performed.

## DISCUSSION

**Fig. 6.**
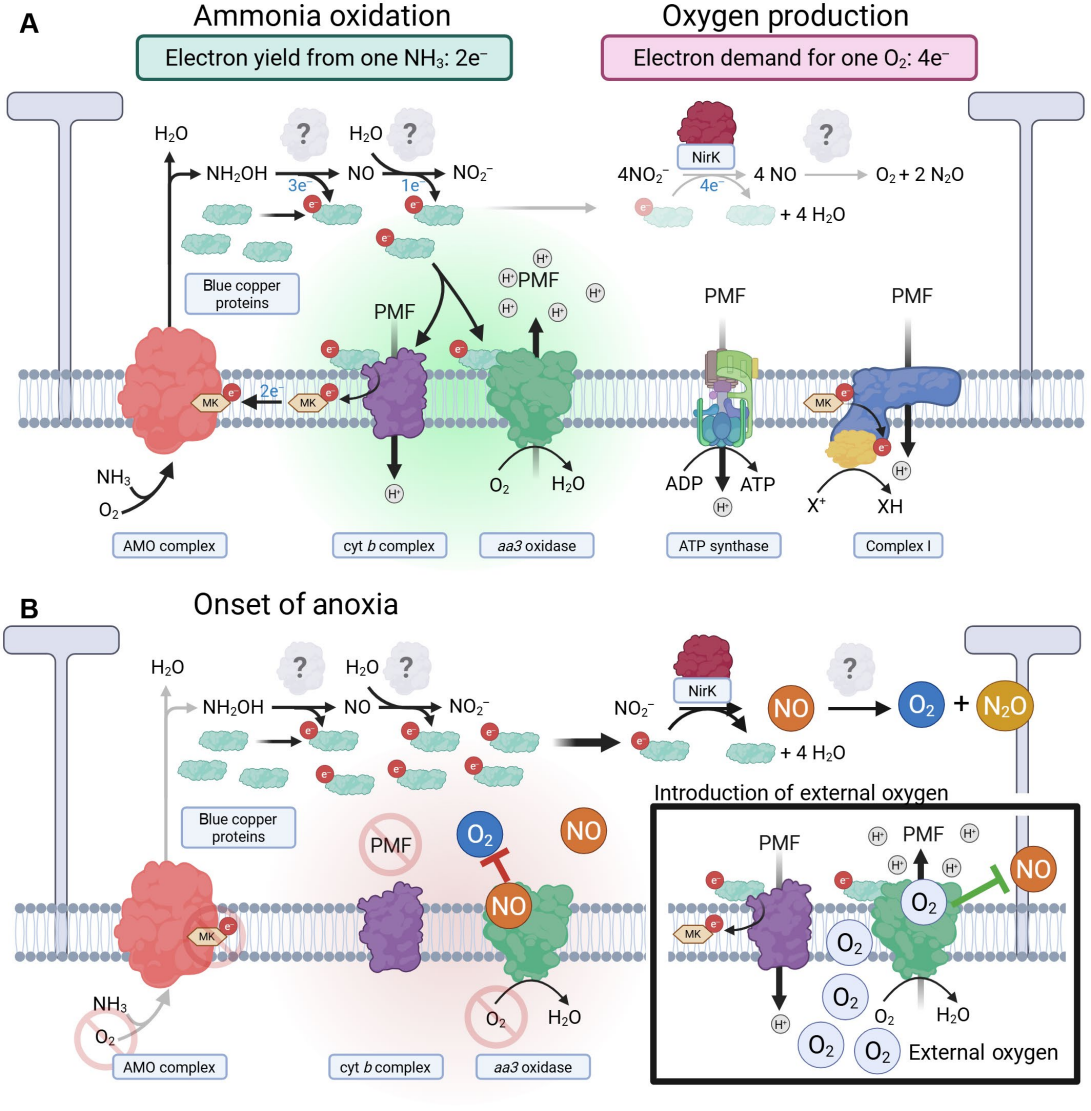
Ammonia oxidation and oxygen accumulation under oxic and anoxic conditions. (**A**) Depiction of ammonia oxidation under normal oxic conditions. Electrons extracted from the oxidation of hydroxylamine to nitrite are transferred to blue copper proteins and, ultimately, are used by the terminal oxidase (aa_3_oxidase) to create a proton motive force (PMF). The produced PMF is used for energy production, the reduction of the quinone pool, and the introduction of electrons into the cell. Oxygen production is only used to prevent a buildup of reduced blue copper proteins and reactive intermediates. (**B**) Under anoxia, the PMF collapses because of the lack of oxygen at the terminal oxidase halting all PMF-dependent processes and preventing the activation of NH_3_ by the AMO complex. Reduced blue copper proteins are redirected toward nitrite reductase (NirK), resulting in the production of nitric oxide, oxygen, and nitrous oxide as electron sinks to prevent the buildup of toxic intermediates. Produced oxygen is not used because of competitive inhibition at the terminal oxidase with nitric oxide. The black box represents the influence of external oxygen that displaces nitric oxide and allows the cell to function normally until again running out of oxygen. MK, menaquinone. Created in BioRender. L. Hodgskiss (2026), https://BioRender.com/9gb9c5r.

Ammonia oxidation in AOA relies on oxygen. Oxygen is consumed by the AMO complex and the terminal oxidase, generating a proton motive force (PMF) that fuels the production of adenosine 5′-triphosphate (ATP) via activity of ATP synthase. Although the specific pathway by which electrons reach the terminal oxidase remains unknown, electrons must also be transferred into the menaquinone pool, which, in turn, supplies the reducing power required for AMO activity, tightly coupling oxygen consumption, energy conservation, and substrate oxidation (Fig. 6). Thus, all central processes in archaeal ammonia oxidation are intrinsically dependent on oxygen availability.

### Oxygen production as an electron overflow pathway

Kraft *et al.* (*29*), who first observed oxygen production in marine AOA, suggested that it can sustain ammonia oxidation activity under anoxic conditions, with most of the produced oxygen consumed internally and only a small excess detectable externally (*29*), similar to dark oxygen production in “*Candidatus* Methylomirabilis oxyfera” (*42*). This was supported by an increase in oxygen accumulation after inhibition of the terminal oxidase by cyanide, i.e., the terminal oxidase was concluded to be actively consuming produced oxygen. Additionally, small levels of N^15^ labeled nitrite were also found to be produced from supplied N^15^ ammonia during oxygen production.

In this study, the ability of AOA to sustain ammonia oxidation while producing oxygen was evaluated by removing NH_4_^+^ from the system to prevent activity of the AMO complex directly. This was made possible by comparing oxygen consumption/production patterns under specific conditions. To discount the possibility of internal NH_4_^+^ storage, oxygen production was also evaluated in the presence of ATU, an inhibitor of nitrifiers that has recently been shown to specifically target the AMO complex in *N. viennensis* (*10*, *41*). The notably similar oxygen production patterns with or without ammonia shown here (Fig. 1) make the simultaneous consumption and production of oxygen, where most oxygen is consumed for ammonia oxidation, unlikely. If oxygen production primarily sustained ammonia oxidation activity under anoxic conditions, then incubations without ammonia or with ammonia in the presence of ATU should have produced substantially more oxygen. Instead, a slightly higher total production without NH_4_^+^ present in the medium (Figs. 1 and 5) implies a low level/negligible activity of the AMO. This difference in total production, corresponding to the amount of oxygen consumed for ammonia oxidation, [Fig. 5 (i and v)], indicates that oxygen production in this setup sustained only minor AMO activity (dataset S1).

It has also been hypothesized that oxygen production may give an advantage to the cell. However, rather than conferring a physiological benefit, oxygen production impaired recovery from anoxia (Fig. 2). This disadvantage may arise because oxygen production consumes electrons from the pool of available reducing equivalents, likely reduced blue copper proteins (*14*), to reduce NO_2_^−^. At the beginning of all experiments with NO_2_^−^, the remaining oxygen is quickly consumed (Figs. 1, 3, and 4). Once depleted, the activity of the terminal oxidase (complex IV) ceases, collapsing the PMF. Without a PMF, electrons can no longer enter the membrane. In this scenario, reduced blue copper proteins require an alternative oxidation route.

To address this, it is hypothesized that NO_2_^−^ reduction acts as an electron overflow pathway, alleviating nitrosative stress from NH_2_OH accumulation [that requires oxidized electron acceptors (i.e., blue copper proteins) for the transfer of electrons], while balancing the redox state to maintain efficient ammonia oxidation (Fig. 6). This pathway would alleviate stress by accepting electrons from blue copper proteins to reduce nitrite to NO, relieving this bottleneck. However, NO can react abiotically with NH_2_OH to form N_2_O, representing an uncontrolled side reaction that dissipates the critical intermediate (i.e., NH_2_OH) (*43*, *44*). Therefore, NO must also be removed from the system to prevent unwanted reactions and alleviate nitrosative stress. The proposed NO dismutation reaction may, therefore, serve as a detoxifying function by preventing the accumulation of NO. Under oxic conditions, these reactions would be a way to respond quickly to changes in reactive intermediates.

In this scenario, NO_2_^−^ reduction could occur concurrently with NH_2_OH accumulation, and the interaction between NO and NH_2_OH may unavoidably lead to enhanced nonenzymatic N_2_O formation. This would be particularly enhanced if oxygen availability is low, leading to both an accumulation of NH_2_OH and a reduction of NO_2_^−^ to NO. This provides a mechanistic explanation for the increased N_2_O yields observed at low oxygen concentrations, facilitated by biotic and abiotic interactions, in the presence of NO_2_^−^, both in laboratory cultures (*20*) and environmental systems (*45*, *46*). In anoxic conditions with reduced blue copper proteins present, NO_2_^−^ reduction would remain as the sole route for electrons leading to a buildup of the products of electron overflow, mainly NO and oxygen (Fig. 6).

This electron-dependent interpretation of oxygen production matches the observations that starvation, which would decrease the pool of reduced blue copper proteins and, therefore, electron availability, diminished oxygen production capacities [Figs. 3 and 5 (i and vii)]. Although starvation may also affect overall metabolic activity or cell viability, *N. viennensis* is known to easily recover from much longer periods of starvation (*47*), and, therefore, a significant decrease in cell viability was discounted.

The dynamic effect of recent ammonia oxidization activity influencing oxygen production becomes further evident, when additions of oxygen allow AMO activity to replenish the electron pool in the presence of ammonia (Fig. 4A). In this case, significantly more total oxygen was produced [Fig. 5 (viii)] compared to conditions that had NH_4_^+^ with little [Fig. 5 (ix)] or no [Fig. 5 (i)] added oxygen, indicating a limitation that is alleviated by the presence of external oxygen. This limitation may be the availability of reduced blue copper proteins (Fig. 4A) that can be replenished in the presence of both ammonia and externally added oxygen. In contrast, when oxygen additions could not replenish the electron pool (Fig. 4, B and C), either because no NH_4_^+^ was available [Fig. 5 (x)] or the AMO was inactivated by ATU [Fig. 5 (xii)], the total production stayed constant compared to control conditions with no oxygen additions and no NH_4_^+^ [Fig. 5 (v)], i.e., the introduction of external oxygen no longer leads to a significant increase in total oxygen production. This is presumably due to an inability of the AMO complex to use external oxygen to replenish the electron pool due to either the absence of NH_4_^+^ or inhibition by ATU.

The proposed function as an electron balance pathway would serve an identical function as that of nitrifier denitrification in AOB, where nitrite reduction serves as an electron sink from the cytochrome pool during aerobic ammonia oxidation (*33*, *48*, *49*), and would also be analogous to heterotrophic denitrification by bacteria or fungi (*50*, *51*). Comparable nitrite-based electron sinks have also been described in cyanobacteria (*52*, *53*). NirK has been proposed for several roles in the core metabolism of ammonia oxidizers, either facilitating a reverse reaction of NO to NO_2_^−^ (*12*, *54*) or as a NirK (*55*), where resulting NO would be involved in the oxidation of NH_2_OH (*13*). However, the absence of NirK from the core genome of AOA (*6*), especially in the genus *Nitrosocaldus* (*56*, *57*), makes an involvement in core metabolism unlikely. Instead, if NirK functions in an electron overflow pathway, then this would imply constitutive expression to rapidly respond to the buildup of intermediates. This interpretation is consistent with its constant and high expression levels in many natural environments (*54*, *58*–*60*), axenic laboratory cultures, and various stress conditions (*61*–*63*). Under oxic conditions, oxygen production at these levels would remain undetectable. However, because different AOA strains yield either N_2_O or N_2_, this pathway could act as either a sink or a source of N_2_O (*30*), even under oxic conditions. Thus, under oxic conditions, this pathway alleviates redox stress from reactive intermediates, but, under anoxia, it becomes a futile process, accumulating NO and subsequently N_2_O and O_2_.

Due to the absence of NirK in the core genome of AOA, it is possible that not all AOA strains use this electron overflow pathway and, therefore, might not produce oxygen in a nitrite dependent manner under anoxia. This opens the possibility that clades missing NirK (i.e., *Nitrosocaldus*) use a different mechanism for regulating their electron pool. Future studies, particularly those involving NirK-deficient strains, may help to more conclusively resolve the involvement of NirK. The enzyme responsible for NO dismutation that follows this step remains unknown and is now indistinguishable from the core genome of AOA but may be identifiable through comparative genomics if an AOA strain incapable of oxygen production is identified.

It should also be noted that, thus far, all cultivated strains that have been tested for oxygen production have been isolated from aerobic environments and are strictly dependent on ammonia oxidation coupled to oxygen reduction for growth. Therefore, it is also possible that specialized lineages in anoxic habitats may have repurposed this unique pathway to sustain activity under anoxic conditions by using other electron donors to fuel this process. This would be in line with the presence of strictly aerobic organisms in permanently anoxic sediments (*64*), where dark oxygen production might sustain oxygen dependent metabolism (*65*).

### Why is produced oxygen not consumed?

If oxygen production functions as an electron sink during aerobic oxidation, then strictly anoxic conditions do not represent the default state of this phenomenon. Nevertheless they yield informative insights. Under anoxia, similar oxygen production is observed regardless of whether the AMO complex is active, inhibited by ATU, or lacks a substrate, underscoring the negligible activity of AMO during oxygen production. This raises the question of why the generated oxygen is not used. If this pathway is the sole electron sink under these conditions, then even trace amounts of produced oxygen should be consequently consumed via the terminal oxidase and AMO. In contrast to this assumption, oxygen accumulation is observed (Fig. 1), suggesting that the organism cannot use the self-produced oxygen. Unexpectedly, exogenous oxygen additions trigger consumption of oxygen even when they are supplied below the production threshold (Fig. 4). Additionally, larger oxygen additions resulted in higher consumption (Fig. 4A). Once consumption started, even endogenously accumulated oxygen that had previously remained unused was depleted. Higher oxygen conditions led to a full consumption of oxygen, upon which production started again, whereas lower additions led to a partial consumption, with production resuming at a concentration determined solely by the amount of oxygen added (Fig. 4). A similar pattern has also been described by Hernández-Magaña *et al*. (*30*) and was interpreted as being actively regulated by the cell. Active regulation could be achieved by transcriptional and translational control or through the sensing of different oxygen concentrations. Transcriptional control seems unlikely because the transcriptome of *N. viennensis* showed signs of degradation within the first 2 hours of oxygen production, well before the bulk of oxygen was produced (dataset S1 and figs. S1 to S4). Additionally, a change in the proteome in such a short time frame is unlikely based on recent evidence that proteome changes are not detected until 6 to 12 hours after the induction of stress in *N. viennensis* (*41*). However, proteins that are already present could still respond to environmental changes. Even so, a regulatory switch based on oxygen concentration also seems unlikely as different behaviors are observed at the same oxygen level; the primary distinction is that consumption is triggered when oxygen is supplied exogenously. If concentration is not the determining factor, then an additional element must be involved. This points to the possibility that oxygen consumption is competitively inhibited until a higher amount of oxygen is introduced to displace the inhibitor. Given the central role of the terminal oxidase in oxygen reduction, NO emerges as a compelling candidate.

The current model of oxygen production is dependent on the reduction of NO_2_^−^ to produce NO. Fitting this model, NO accumulation has been observed in all ammonia oxidizers during oxygen production, typically reaching concentrations up to 10-fold higher than O_2_ (*29*, *30*). NO can competitively and reversibly bind to the terminal oxidase, inhibiting its activity at nanomolar concentrations in an O_2_ and NO-dependent manner (*66*, *67*). Exogenous O_2_ additions may shift the O_2_:NO ratio in favor of O_2_, displacing NO from the terminal oxidase. Larger O_2_ additions have a stronger effect on this ratio, thereby enhancing O_2_ consumption (Fig. 4A). Consistent with this mechanism, NO concentrations have been shown to drop following O_2_ additions, after which both NO and O_2_ increased in parallel (*30*). The disappearance of NO after exogenous oxygen addition is consistent with NO itself being an intermediate in the ammonia oxidation process (*12*, *15*); once external oxygen is added and displaces NO, the cell functions as normal until oxygen is again depleted. This offers a plausible explanation for the seemingly counterintuitive lack of a threshold for onset of production or consumption. During undisturbed production, NO and O_2_ accumulate together, resulting in higher, unused O_2_ concentrations. The added oxygen, however, changes this ratio, disassociates inhibition, and allows consumption by the terminal oxidase and, subsequently, also AMO activity, which could alternatively also be directly inhibited by NO. A comparable role of NirK-derived NO in regulating cellular redox balance has been proposed for *Nitrobacter winogradskyi*, where NO production is suggested to modulate electron flow through reversible inhibition of the terminal oxidase under low-oxygen conditions (*68*). This conceptual parallel supports a broader role for NO as a regulator of electron flow in nitrifiers. A current unknown in this scenario is the reactivity of NO with the terminal oxidase in AOA. While NO is not reduced in some eukaryotic oxidases, the terminal oxidase of *Thermus thermophilus* has been shown to catalyze the reaction of two NO to N_2_O (*69*), adding another potential source of N_2_O. While this might account for some of the N_2_O produced in AOA cultures, it would not account for the production of oxygen or the continued reduction to N_2_ observed in some strains.

### Reconciling oxygen production with ammonia oxidation

Oxygen production has emerged as a shared feature among diverse AOA, yet its physiological role has remained unclear. Because it requires more electrons than ammonia oxidation can provide, it cannot sustain metabolism under anoxic conditions. Instead, the results presented here suggest that oxygen production acts as an electron overflow mechanism, alleviating nitrosative stress and maintaining redox balance during normal growth, as is also seen for nitrifier denitrification in AOB (*48*, *49*). In line with this, other detoxification mechanisms such as MCO4a have also been proposed (*70*), which is not unexpected given that organisms with such a highly reactive core metabolism require multiple safeguards. This perspective also resolves several apparent contradictions of the earlier model (*29*, *30*, *35*): the lack of sustained activity under anoxia, the reduced recovery once electron pools are depleted, and the seemingly counterintuitive reduction of N_2_O to N_2_, which could act as another electron sink. The high oxygen concentrations observed here under strictly anoxic laboratory conditions, therefore, represent the product of a detoxification pathway out of its natural, oxic context and are unlikely to occur in the environment. This is consistent with previous observations that NO_2_^−^ is reduced to NO under oxic conditions in *N. maritimus*, indicating that NO_2_^−^-derived NO can contribute to N_2_O production alongside NH_3_-derived intermediates (*34*). Under normal oxic conditions, this contribution would be quite low as an accumulation of reduced blue copper proteins is unlikely. Rather, N_2_O is produced predominantly from the core ammonia oxidation process (*34*) and the unavoidable abiotic reaction of some intermediates (*13*). Under anoxic conditions, the core ammonia oxidation process is slowed, and a buildup of reduced blue copper proteins is dealt with via the reduction of NO_2_^−^ to NO. This would be followed by either the production of O_2_ and N_2_O by an unknown enzyme or the abiotic reaction of NO with other intermediates to form N_2_O. In a scenario such as this, N_2_O from AOA would predominantly be derived from the NirK-facilitated reduction of NO_2_^−^ to NO. Last, the finding that most self-produced oxygen is not consumed can be explained by the reversible inhibition of the terminal oxidase by NO (*66*, *67*), which is released once external oxygen is supplied. Together, these findings establish oxygen production in AOA not as an adaptation to anoxic conditions, but as a redox balancing strategy that safeguards cells under nitrosative stress.

## MATERIALS AND METHODS

### General cultivation and preparation of anoxic medium

#### Cultivation

*N. viennensis* EN76 (*32*) was cultivated as previously described (*32*). Basic freshwater medium (FWM) was supplemented with 2 mM NH_4_Cl, 2 mM NaHCO_3_, 7.5 μM FeNaEDTA, 1 mM pyruvate, kanamycin (50 μg/ml), and nonchelated trace elements [0.1% volume/volume (v/v)] and buffered with 10 mM Hepes to pH 7.5. All solutions were sterilized by either filtration (0.2-μm mixed cellulose ester membrane filters) or autoclaving (FWM). Nitrite production was followed by colorimetric measurements via the Griess reaction, as described previously. Cultures were incubated at 42°C, shaking at 80 rpm, in the dark. Cell numbers were determined as previously described, by linking NO_2_^−^ values to cell counts (*47*) (https://github.com/pribasnig/N_viennensis_NO2_to_cellnumber_tool) and available on Zenodo (https://zenodo.org/records/20110940, DOI: 10.5281/zenodo.20110940). Scripts are available on GitHub (https://github.com/pribasnig/oxygen_production) and Zenodo (https://zenodo.org/records/20110874, DOI: 10.5281/zenodo.20110874).

For experiments, cells were concentrated by centrifugation in a Sorvall Lynx 400 centrifuge using a Thermo Fisher Scientific Fiberlite F12-6x500 LEX rotor and 400-ml centrifugation vessels (21,000*g*, 30 min, 40°C), washed in NH_4_Cl-free growth medium to remove residual NH_4_Cl or NO_2_^−^ and centrifuged again. Cell pellets were resuspended in a known volume of NH_4_Cl-free growth medium, pooled, and distributed into the respective experimental conditions. The amounts of cells used were similar over all experiments (7.0 × 10^9^ ± 6.8 × 10^8^ cells, dataset S1). Cell-specific oxygen production rates (in attomoles per cell) were calculated by dividing net production (in micromoles) by cell numbers (dataset S1).

#### Anaerobic preparations

Serum bottles (120 ml) were equipped with PreSens PSt6-YAU-D5-YOP oxygen sensor spots, following the manufacturer’s instructions, autoclaved twice, and filled with 80 ml of respective medium under sterile conditions. Bottles were sealed airtight using 20-mm blue butyl rubber stoppers and 20-mm aluminum crimp caps to seal the bottles, then sparged with N_2_ for 30 min, incubated overnight at 42°C (80 rpm), and sparged again for 30 min. Oxygen sensors were factory calibrated before use. Oxygen concentrations were measured with a Fibox 4 trace optode. If concentrations were >0.5 μM, then flasks were sparged further until concentrations of ≤0.5 μM were reached. As nitrogen gas may contain trace amounts of oxygen, all preparations are referred to as near anoxic. Near-anoxic flasks were transferred to an anaerobic chamber (Coy Laboratory Products, Grass Lake, USA) with a 98% N_2_ and 2% H_2_ atmosphere and a palladium catalyst, where they were completely filled with near-anoxic medium prepared the same way, using sterile syringes and needles, leaving only a small N_2_ bubble for incubations later. Bottles were transferred to 42°C for at least 24 hours before experiments were started and O_2_ concentrations measured again. All conditions contain 2 mM NH_4_^+^ and 1 mM NO_2_^−^, unless otherwise specified.

#### Incubations with different amounts of NO_2_^−^ and NH_4_^+^

To test the dependence on NO_2_^−^, near-anoxic medium was prepared with different concentrations of NO_2_^−^ (0, 0.05, 1, or 10 mM). Substrates were added either individually or in combination, as specified for the respective experiments below. To assess the influence of NH_4_^+^, near-anoxic medium was prepared with either 2 or 0 mM NH_4_Cl. In each treatment, 0.5 ml of concentrated cells were inoculated into the medium and maintained in a water bath at 42°C, and oxygen consumption and production monitored over 24 hours. Cell numbers for each experiment can be found in dataset S1.

#### Recovery after anoxic conditions

After 24 hours of anoxic incubation, cultures either oxygen producing (1 mM NO_2_^−^ and 2 mM NH_4_Cl) or non–oxygen producing (0 mM NO_2_^−^ and 2 mM NH_4_Cl) were returned to optimal oxic growth conditions (0 mM NO_2_^−^ and 2 mM NH_4_Cl). For this, bottles were opened sterilely, and 1 ml (6.12 × 10^7^ cells) was inoculated immediately into 20 ml of 42°C prewarmed, optimal growth medium in 30-ml polystyrene containers (Greiner Bio-One, G201170). Growth was followed over time via nitrite measurements as described above.

#### Starvation experiments

Cells were resuspended and pooled as described above and then divided into three groups. The first group was immediately inoculated into near-anoxic medium containing 1 mM NO_2_^−^ and 2 mM NH_4_Cl. The second and third groups were incubated in 20 ml of NH_4_Cl-free medium at 42°C for 24 or 72 hours, respectively, to starve cells. After those incubations, starved cells were centrifuged, resuspended, and inoculated into near-anoxic medium containing 1 mM NO_2_^−^ and 2 mM NH_4_Cl. Oxygen production was monitored over 24 hours in all treatments.

#### Oxygen additions

To test the effect of oxygen additions, cells were inoculated into near-anoxic medium containing 1 mM NO_2_^−^ under three conditions: condition 1 with 2 mM NH_4_Cl, condition 2 with 0 mM NH_4_Cl, and condition 3 with 2 mM NH_4_Cl and 3 mM ATU. In condition 1, replicates were split into two groups. When oxygen was produced to a concentration of ∼1 μM, the first group received an addition of 0.5 ml of 42°C oxic growth medium (containing 98.5 nmol of oxygen), leading to an O_2_ increase of 0.82 μM, and the second group received 1 ml (containing 197 nmol of oxygen), leading to an O_2_ increase of 1.64 μM. For the second group, the same additions (containing 197 nmol of oxygen), leading to an O_2_ increase of 1.64 μM each time, were repeated twice more, at concentrations of 1 and 1.4 μM.

Condition 2 replicates were split in two groups. No additions were made to the first group. The second group received two additions of 1 ml of 42°C oxic growth medium (containing 197 nmol of oxygen), leading to an increase of 1.64 μM for each addition. Additions were made when oxygen was produced to concentrations of ∼1 μM.

Condition 3, replicates were split into two groups. After oxygen was produced to a concentration of ∼0.8 μM, one group received an addition of 1 ml of 42°C anoxic medium, the other group 1 ml of 42°C anoxic medium containing 345 mM ATU, resulting in a final concentration of 3 mM ATU. Additions of anoxic medium or anoxic medium containing ATU did not influence oxygen production (Fig. 3C, gray arrow). When oxygen was produced to a concentration of ∼1.5 μM, both groups received additions of 1 ml of 42°C oxic growth medium (containing 197 nmol of oxygen; Fig. 3C, black arrow), increasing concentrations by 1.64 μM. In all experiments, oxygen concentration was measured at varying time intervals to determine consumption and production over 24 hours.

#### Transcriptome analysis

To conduct transcriptomic studies, *N. viennensis* was grown in 500 ml of optimal oxic medium. Upon reaching mid-exponential phase, cultures were either directly filtered to use as a control or centrifuged and inoculated in near-anoxic medium, where oxygen production was monitored. To harvest cultures after 24 hours under oxygen producing conditions (1 mM NO_2_^−^ and 2 mM NH_4_Cl), cultures were opened and filtered in an anaerobic chamber (Coy Laboratory Products, Grass Lake, USA) with a 98% N_2_ and 2% H_2_ atmosphere and a palladium catalyst, and filters were stored in Falcon tubes, flash frozen, and incubated at −70°C. RNA was extracted using the mirVana miRNA Isolation Kit (Thermo Fisher Scientific) according to the manufacturer’s instructions and as described before (*61*). RNA was submitted to the Vienna Biocenter Facility (VBC) for sequencing with a species-specific rRNA depletion step. Samples from the 24-hour experiment were sequenced as 100–base pair single-end readsRaw reads were downloaded from VBC and checked with md5sum. FastQC v.0.12.1 (*71*) and multiQC v.1.25 (*72*) were used to analyze the reads. Reads were trimmed using fastp v.0.23.4 (*73*) with specific settings: rim_front1 12 --trim_front2 12 --trim_poly_g --detect_adapter_for_pe --average_qual 30 --length_required 30. SortmeRNA v.4.3.4 (*74*) was used to sort out rRNA reads and identified mRNA reads were mapped to the *N. viennensis* reference genome and associated annotation datasets, downloaded from NCBI (www.ncbi.nlm.nih.gov/datasets/genome/GCF_000698785.1/), using HISAT2 v.2.2.1 (*75*). Genome indexing and mapping were performed with the corresponding genomic FASTA nucleotide file (.fna) and the .gff file from the same GCF assembly. Reads were treated as single-end, and library strandedness was specified during alignment (--rna-strandness R). Gene-level counts were obtained using featureCounts v2.1.1 (*76*) and the associated general feature format file (gff) associated with the aforementioned genome. To calculate TPM values, the read counts were divided by the length of each gene in kilobases, which yielded reads per kilobase (RPK). The sum of all RPK values was divided by 1,000,000, giving a per-million scaling factor, which was used to divide individual RPK values to yield TPM. Because the anoxic transcriptomes were degraded, differential expression analysis was not carried out.

After degradation (figs. S1 to S4) was observed, the experiment was repeated in a shorter time frame. This time cells were harvested after 2 hours under oxygen producing conditions. All methods used were identical to the 24-hour experiment, with the exception that samples from the 2-hour experiment were sequenced as 150–base pair paired-end reads, and reads were treated as paired-end reads with library strandedness specified during alignment (--rna-strandness RF). All scripts used for transcriptomic analysis are available on GitHub (https://github.com/pribasnig/oxygen_production) and Zenodo (https://zenodo.org/records/20110874, DOI: 10.5281/zenodo.20110874).

#### Statistical methods

Total produced oxygen data were plotted for each condition after 24 hours (dataset S1). Data were tested in R version 4.5.1 (*77*) using Rstudio 2025.09.1.401 (*78*) for normality and homogeneity of variance using the Shapiro test [shapiro_test, car package v3.1-3; (*79*)] and Levene test [leveneTest, rstatix v0.7.3 package; (*80*)], respectively. While all conditions passed the normality check, the assumptions for homogeneity of variance were not met. Therefore, conditions were evaluated for differences in means using the Games-Howell test [GamesHowellTest, PMCMRplus v1.9.12 package; (*81*)], which is suitable for data with unequal variances. Post hoc analysis [posthocTGH, rosetta v0.3.12 package; (*82*)] was used to obtain adjusted *P* values using the Benjamini-Hochberg method, and conditions were placed into statistically similar groups using the multcompLetters() function of the multcompView v0.1-10 package (*83*). The R package tidyverse v2.0.08 was used for helpful processing of data (*84*).
