# Supplementary material for: Oxygen production as an electron overflow pathway in ammonia-oxidizing archaea
Source: Sci Adv. 2026 Aug 14;12(33):eaee5234. doi: 10.1126/sciadv.aee5234 (PMC13475601; doi:10.1126/sciadv.aee5234)
Supplement: Supplementary file 1 [file sciadv.aee5234_sm.pdf]

Science Advances
AAAS

Supplementary Materials for

Oxygen production as an electron overflow pathway in ammonia-oxidizing archaea

Thomas Pribasnig *et al.*

Corresponding author: Logan H. Hodgskiss, logan.hodgskiss@univie.ac.at

Sci. Adv. **12**, eaee5234 (2026)
DOI: 10.1126/sciadv.aee5234

The PDF file includes:

Figs. S1 to S4
Legend for dataset S1

Other Supplementary Material for this manuscript includes the following:

Dataset S1

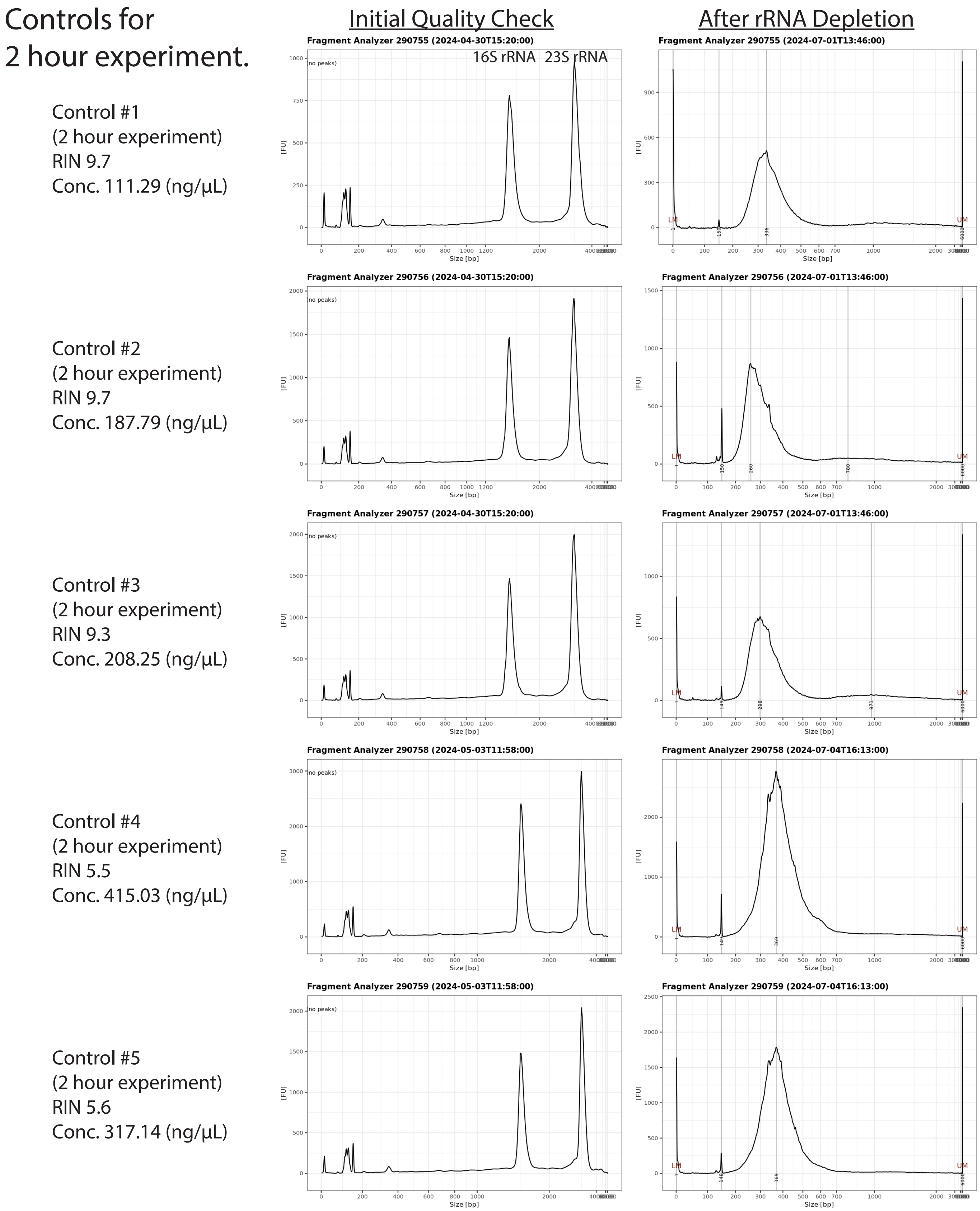

Figure S1: Bioanalyzer data for controls of 2 hour anoxia experiment. Abbreviations: RIN, RNA integrity number.

Samples producing oxygen for 2 hours.

Oxygen Production-Sample #1
(2 hour experiment)
RIN 6.1
Conc. 304.17 (ng/μL)

Oxygen Production-Sample #2
(2 hour experiment)
RIN 5.3
Conc. 203.15 (ng/μL)

Oxygen Production-Sample #3
(2 hour experiment)
RIN 5.3
Conc. 209.06 (ng/μL)

Oxygen Production-Sample #4
(2 hour experiment)
RIN 8.3
Conc. 126.19 (ng/μL)

Oxygen Production-Sample #5
(2 hour experiment)
RIN 5.3
Conc. 131.73 (ng/μL)

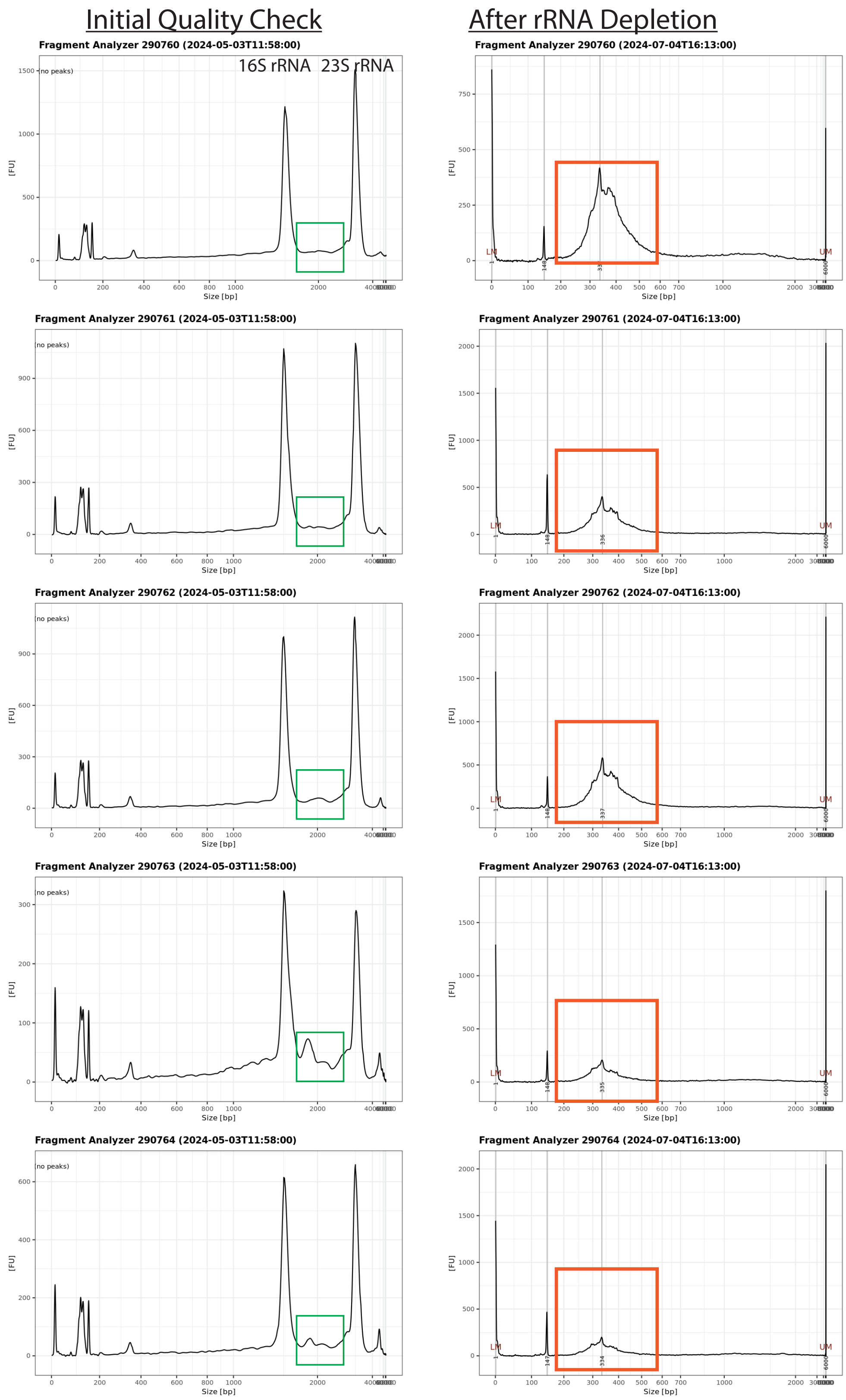

Figure S2: Bioanalyzer data for samples of 2 hour anoxia experiment. Abbreviations: RIN, RNA integrity number. Green boxes in the initial quality check indicate small peaks between the 16S and 23S rRNA peaks that are not found in control samples and could be indicative of degradation. Orange boxes highlight RNA peaks after rRNA depletion that are much smaller and more jagged than control peaks, likely due to RNA that has begun to be degraded.

Controls for 24 hour experiment.

Control #1
(24 hour experiment)
RIN 9.1
Conc. 133.60 (ng/µL)

Control #2
(24 hour experiment)
RIN 8.8
Conc. 152.12 (ng/µL)

Control #3
(24 hour experiment)
RIN 9.4
Conc. 189.62 (ng/µL)

Control #4
(24 hour experiment)
RIN 9.3
Conc. 122.88 (ng/µL)

Control #5
(24 hour experiment)
RIN 9.3
Conc. 74.72 (ng/µL)

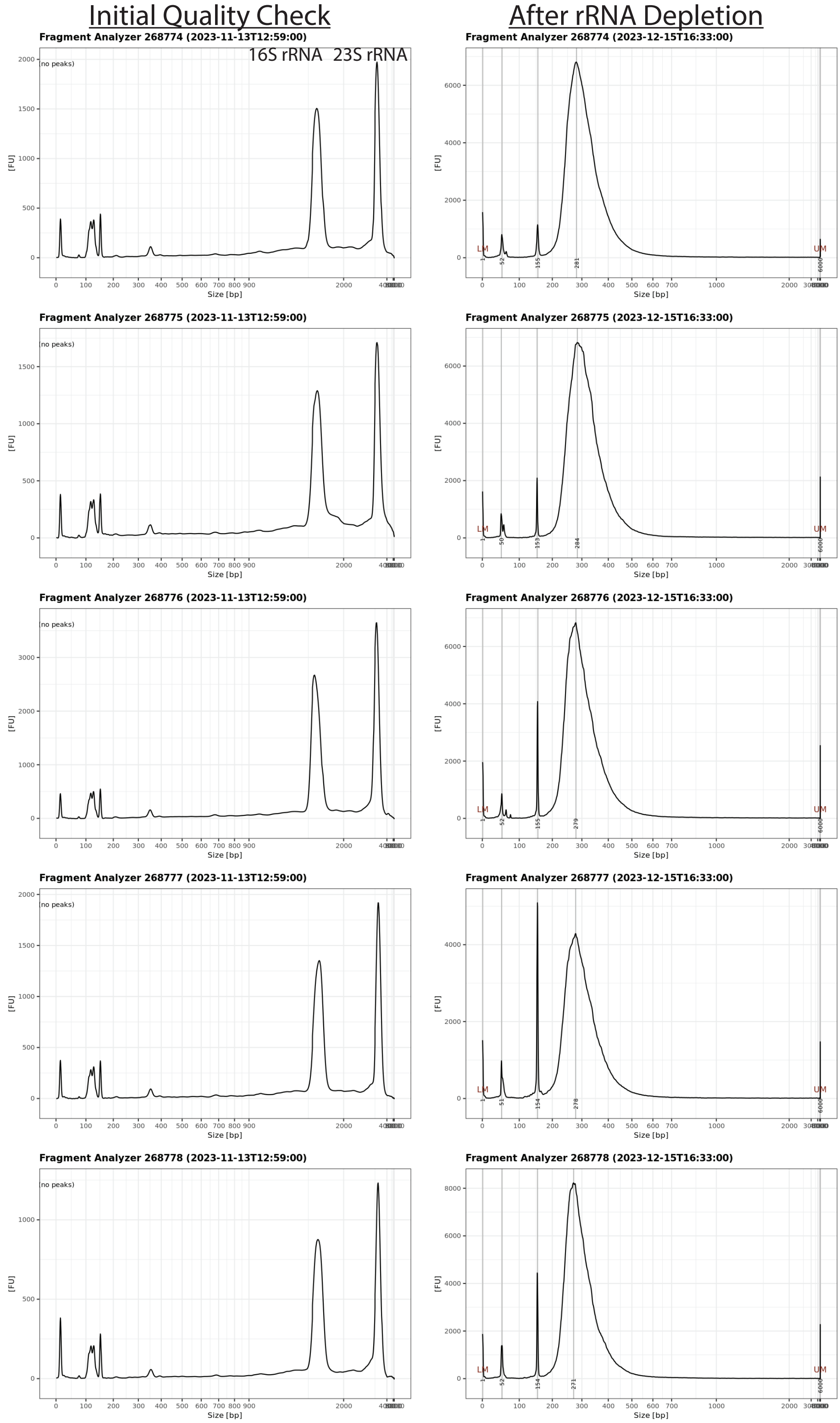

Figure S3: Bioanalyzer data for controls of 24 hour anoxia experiment. Abbreviations: RIN, RNA integrity number.

Samples producing oxygen for 24 hours.

Oxygen Production-Sample #1
(24 hour experiment)
RIN 8.3
Conc. 168.62 (ng/µL)

Oxygen Production-Sample #2
(24 hour experiment)
RIN 8.5
Conc. 179.23 (ng/µL)

Oxygen Production-Sample #3
(24 hour experiment)
RIN 7.7
Conc. 261.10 (ng/µL)

Oxygen Production-Sample #4
(24 hour experiment)
RIN 8.3
Conc. 176.94 (ng/µL)

Oxygen Production-Sample #5
(24 hour experiment)
RIN 8.5
Conc. 283.16 (ng/µL)

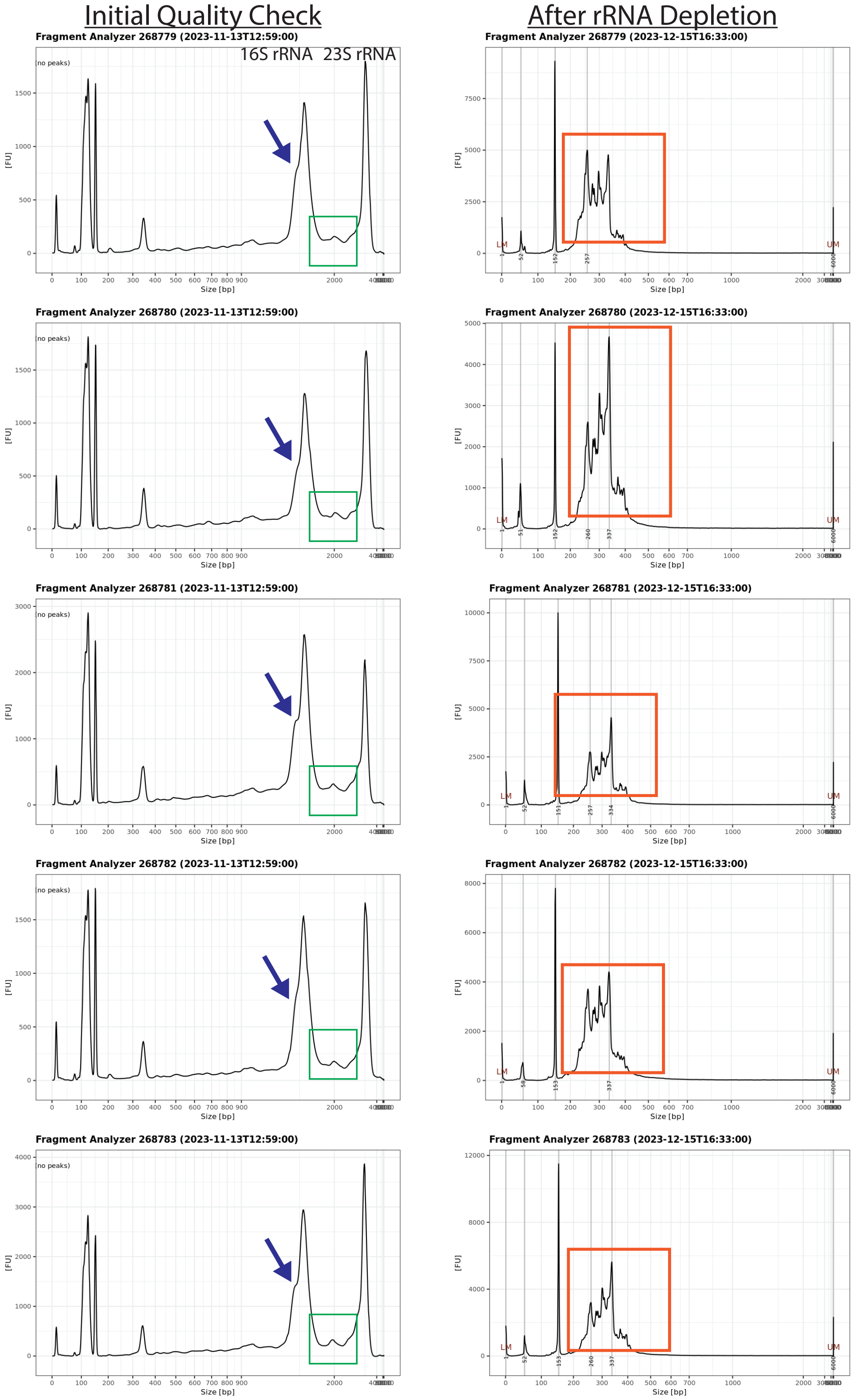

Figure S4: Bioanalyzer data for samples of 24 hour anoxia experiment. Abbreviations: RIN, RNA integrity number. Green boxes in the initial quality check indicate small peaks between the 16S and 23S rRNA peaks that are not found in control samples and could be indicative of degradation. Blue arrows in the initial quality check indicate a shoulder peak in the 16S signal that is strongly suggestive of the degradation of 16S rRNA. Orange boxes highlight RNA peaks after rRNA depletion that are much smaller and more jagged than control peaks, likely due to RNA that has begun to be degraded.

Supplementary Dataset S1
Excel sheet including growth data for all figures and transcriptomic datasets.
